# Diversity of *Akanthomyces* on moths (Lepidoptera) in Thailand

**DOI:** 10.3897/mycokeys.71.55126

**Published:** 2020-07-30

**Authors:** Arifah Nur Aini, Suchada Mongkolsamrit, Wijanarka Wijanarka, Donnaya Thanakitpipattana, J. Jennifer Luangsa-ard, Anto Budiharjo

**Affiliations:** 1 Department of Biology, Faculty of Science and Mathematics, Diponegoro University, Jl. Prof. Sudharto SH, Semarang 50275, Indonesia; 2 Plant Microbe Interaction Research Team, National Center for Genetic Engineering and Biotechnology (BIOTEC), 113 Thailand Science Park, Phahonyothin Road, Khlong Nueng, Khlong Luang, Pathum Thani 12120, Thailand; 3 Biotechnology Study Program, Faculty of Science and Mathematics, Diponegoro University, Jl. Prof. Sudharto SH, Semarang 50275, Indonesia; 4 Molecular and Applied Microbiology Laboratory, Central Laboratory of Research and Service, Diponegoro University, Jl. Prof. Sudharto SH, Semarang 50275, Indonesia

**Keywords:** *
Akanthomyces
*, entomopathogenic fungi, fungal taxonomy, multilocus phylogeny

## Abstract

*Akanthomyces* is a genus of invertebrate-pathogenic fungi from the family Cordycipitaceae (Ascomycota, Hypocreales). Its species occurs on two different types of hosts, spiders and insects, and in the latter case specifically Lepidoptera adults. Three new species of *Akanthomyces*, *A. noctuidarum*, *A. pyralidarum*, and *A. tortricidarum* occurring on adult moths from Thailand are proposed based on the differences of their morphological characteristics and molecular data. Phylogenetic analyses using a combined dataset, including the internal transcribed spacer regions, the large subunit of the ribosomal DNA, translation elongation factor 1-α, the largest subunit of RNA polymerase II, and the second largest subunit of RNA polymerase II, support the delimitation of these new species in *Akanthomyces*.

## Introduction

Cordycipitaceae is one of the families of the order Hypocreales with entomogenous nutritional habit. Many of the species in this family have been originally isolated from dead insects and spiders that are buried in the soil, leaf litter, or attached to the undersides or upper sides of a leaf. Some species, especially in *Beauveria*, could be found in the soil (Rehner and Buckley 2005) or as endophytes (Mantzoukas and Lagogiannis 2019; Afandhi et al. 2019). Cordycipitaceae is validated based on the type of *Cordyceps*, *Cordyceps militaris*, and it has initially included pyrenomycetes that possess pallid to brightly colored, ﬂeshy stromata (Kepler et al. 2017). It is also characterized by producing superficial to completely immersed perithecia, cylindrical asci with thickened apex, and multi-septate filiform ascospores that disarticulate into part-spores or remain intact at maturity (Sung et al. 2007). Well-known for its use in traditional Chinese medicine, *C. militaris* produces some polysaccharides and cordycepin that have been used for anti-inflammatory, antioxidant, anti-tumor, anti-metastatic, and immunomodulatory functions (Das et al. 2010). The recent study of *C. militaris* shows that this fungus has an anti-hypertension and neuroprotective effect to delayed neural death (Takakura et al. 2017; Kim et al. 2018).The most popular anamorph in this family is *Beauveria*, notably with its type species, *Beauveria bassiana*, which has been used globally as a mycoinsecticide since the 1960s (Vega et al. 2012). Spider pathogens are mostly found within Cordycipitaceae (Shrestha et al. 2019). Their anamorph are found in *Akanthomyces*, *Gibellula*, or *Hevansia* (Kepler et al. 2017).

*Akanthomyces* was established by Lebert (1858) with *Akanthomyces aculeatus*, the type species, found on a moth in Europe (Mains 1950). *Gibellula* differs from *Akanthomyces* in the production of aspergillus-like conidiophores and the host range (Kepler et al. 2017). *Gibellula* is only found on spiders, while *Akanthomyces* can be found on both, spiders and insects. *Akanthomyces* was known attacking some insect orders such as Hemiptera (*Akanthomyces lecanii*), Coleoptera (*Akanthomyces neocoleopterorum*), Lepidoptera (*Akanthomyces pistillariiformis*), and Orthoptera (*Akanthomyces fragilis*) (Hodge et al. 2003; Mongkolsamrit et al. 2018; Chen et al. 2020). In general, the host range of *Akanthomyces* for both, teleomorph and anamorph are similar. The genus includes *Cordyceps tuberculata* found on adult moths, which is linked to the anamorph *Akanthomyces pistillariiformis*. *Akanthomyces* has taxonomic priority by date over *Lecanicillium*, one of the anamorphs in Cordycipitaceae with verticillium-like morphologies (Gams and Zare 2001; Kepler et al. 2017). The type species of *Lecanicillium*, *L. lecanii* (*Cephalosporium lecanii*, now regarded as *Akanthomyces lecanii*) is found on lice and scale insects and is known as the anamorph of *Cordyceps confragosa*. On the basis of previous studies on *Akanthomyces* in Thailand, Mongkolsamrit et al. (2018) proposed four new species of *Akanthomyces* on spiders, namely, *A. kanyawimiae*, *A. sulphureus*, *A. thailandicus*, and *A. waltergamsii*. Here, we describe three new *Akanthomyces* species found on adult moths (Lepidoptera) from Thailand based on morphological and molecular studies.

Species complexes or cryptic species are common in the kingdom Fungi. Given the simplicity of the phenotypic characters and the overlap of the size and shapes of important diagnostic features, species in many genera cannot be easily classified and identified. Cryptic species refers to taxa that are morphologically similar, yet evidence has shown that they are on different evolutionary paths as revealed by molecular phylogenetic methods and can only be recognized by their DNA sequences. Entomopathogenic fungi from Thailand are commonly encountered in the forests and constitute a huge number in our collections (Kobmoo et al. 2012; Luangsa-ard et al. 2018; Mongkolsamrit et al. 2018; Tasanathai et al. 2019).

In surveys of entomopathogenic fungi in national parks and community forests, collections of pathogens on adult moths were found on the underside of leaves of dicotyledonous forest plants. The phenotypic characters of the collections in having cylindrical to narrowly clavate synnemata and superficial perithecia scattered on the body and wings of the moth identify them primarily to be members of *Akanthomyces* in Cordycipitaceae, mostly as Akanthomyces cf. tuberculatus. The aims of this study were (1) to elucidate the relationships of these collections to known members of Cordycipitaceae, (2) to uncover hidden species in *A. tuberculatus* species complex, and (3) to describe new taxa to accommodate species diversity in *Akanthomyces*.

## Materials and methods

### Fungal materials and isolation

The specimens used in this study were obtained from BIOTEC Culture Collection (BCC) and BIOTEC Bangkok Herbarium (BBH), Thailand. Fungal specimens were collected from several national parks in Thailand. Soil from the forest floor, leaf litter, undersides, and upper sides of the leaves were scanned for fungal growth on dead insects. Collected specimens were stored in plastic boxes, returned to the laboratory, and examined under a stereo microscope (Olympus SZ61). Isolation from the teleomorphs followed the method described by Luangsa-ard et al. (2018).

Isolation from the anamorphs was carried out using a sterilized inoculation needle to pick the conidia out from sporulating structures and then transfer them on to a PDA plate. These plates were stored in a plastic box chamber at room temperature, left overnight until the conidia germinated, and treated the same way as described in Luangsa-ard et al. (2018).

### Colony growth and morphology

Fungal structures of both, anamorph and teleomorph, such as perithecia, asci, ascospores, synnemata, phialides, and conidia were mounted on glass slides with a drop of lactophenol cotton blue solution. Microscopic measurements of 50 individual fungal structures were obtained using a light microscope (Olympus CX31). Variability was provided as the mean ± standard deviation with absolute minima and maxima in parentheses. Detailed colony descriptions and morphological comparisons of some fungal structures were determined from cultures grown on PDA and OA for 14 days at 25 °C (Mongkolsamrit et al. 2018). The colors of specimens and cultures incubated were described and codified following the Online Auction Color Chart (www.boletales.com/2011/01/new-colour-chart-for-mycologists; abbreviated “OAC” herein). For DNA extraction purposes, starter cultures were grown on PDA for 2 weeks at 25 °C.

### DNA extraction

Genomic DNA was extracted from fungal cultures on PDA using a modified CTAB method (Sung et al. 2001). About 600 µL of CTAB buffer was added to the microcentrifuge tube that contained fungal mycelium, which was ground with pestles and incubated at 65 °C for 1 h. Once the suspension had cooled down, 600 µL of chloroform:isoamyl alcohol (24:1) was added. The supernatant was gently mixed until an emulsion was obtained and centrifuged at 12,000 rpm for 20 min. The aqueous phase was transferred to a new sterile microcentrifuge tube. About 300 µL of cold isopropanol alcohol was added to precipitate DNA and left at -20 °C for 1 h. DNA was then separated from the solution by centrifugation at 4 °C and 12,000 rpm for 20 min. The pellet was washed in 200 µL of 70% cold ethanol and air-dried at room temperature. The DNA pellet was then dissolved in 50 µL of TE buffer (10 mM Tris-HCl pH 8.0 and 1 mM EDTA pH 8.0) (Læssøe et al. 2013). The extracted DNA was stored at -20 °C before amplification (Chen et al. 2018).

### PCR amplification and sequencing

Five nuclear loci regions, namely, internal transcribed spacers 1 and 2 along with the 5.8S rDNA (ITS), large subunit of the ribosomal DNA (LSU), translation elongation factor 1-α (*TEF*), the largest subunit of RNA polymerase II (*RPB1*), and the second largest subunit of RNA polymerase II (*RPB2*), were amplified and sequenced. PCR amplifications were conducted in a 25 µL volume consisting of 1× PCR buffer, 0.4 M betaine, 200 µM of each of the four dNTPs, 1 U Taq DNA polymerase (Thermo Scientific, USA), and 0.2 µM of each primer. The primer pairs used in this study were ITS5 and ITS 4 for ITS (White et al. 1990), LROR and LR5 for LSU (Vilgalys et al. 1994), 983F and 2218R for *TEF* (Rehner and Buckley 2005), CRPB1 and RPB1Cr for *RPB1* (Castlebury et al. 2004), and 5F2 and 7cR for *RPB2* (Liu et al. 1999). PCR amplifications were performed using a BioRad T100 thermal cycler following the procedure described in Luangsa-ard et al. (2005) for ITS and Sung et al. (2001) for the other gene regions. PCR products were visualized by ethidium bromide staining after gel electrophoresis of 4 µL of the product in 1% agarose gel (Luangsa-ard et al. 2004). The PCR products were quantified using a standard DNA marker of known size and weight.

### Sequence alignment and phylogenetic analysis

Each DNA sequence was checked for ambiguous bases and assembled in BioEdit v.7.0.5.3 (Hall 2005). Additional sequences from previous studies (Kepler et al. 2017; Mongkolsamrit et al. 2018) were used as a dataset of taxa in Cordycipitaceae. Multiple sequence alignment was conducted with MUSCLE 3.6 software (Edgar 2004) and manually adjusted. The DNA sequences were compared to sequences in the GenBank database by BLAST search to determine the closest matches with *Akanthomyces*. The final sequence alignment of the combined dataset was used for analyses using maximum parsimony (MP), Bayesian inference, and maximum likelihood to infer their phylogenetic relationships.

MP analysis used PAUP4.0a116 (Swofford 2019), and heuristic searches were performed with 100 replicates of random sequence addition and tree bisection reconnection swapping algorithm. Bootstrap analysis was performed using the MP criterion with 1000 replications. MrModeltest 2.2 (Nylander 2004) was used to choose the best model of DNA substitution that fit the data. MrBayes (Ronquist and Huelsenbeck 2003) was used to determine the Bayesian phylogenetic inference with a general time-reversible plus proportion-invariant plus gamma (GTR+I+G) model of DNA substitution as the best model. Maximum likelihood analysis was performed with RAxML-HPC2 on XSEDE in CIPRES Science Gateway 3.3 (https://www.phylo.org/) using a GTRCAT model of evolution with 1000 bootstrap replicates (Stamatakis 2014).

## Results

### Multilocus phylogeny

A total of 55 new sequences from 11 specimens were obtained in this study (Table 1). ITS sequences were used in a preliminary study to select 11 specimens that represent new species. The combined dataset included 101 taxa and four loci consisting of 3511 bp (LSU 850 bp, *TEF* 1041 bp, *RPB1* 732 bp, and *RPB2* 888 bp). *Purpureocillium lilacinum* in Ophiocordycipitaceae was used as the outgroup for this dataset.

**Table 1.** List of species and GenBank accession numbers of sequences used in this study.

Species	Strain	Host	GenBank accession numbers				
			ITS	LSU	*TEF*	*RPB1*	*RPB2*
*Akanthomyces aculeatus*	HUA186145	–	–	MF416520 ^f^	MF416465 ^f^	–	–
	TS772	Lepidoptera; Sphingidae	KC519371 ^g^	KC519370 ^g^	KC519366 ^g^	–	–
*Akanthomyces araneogenum*	GZUIFDX2 T	*Araneus* sp.	KU893153 ^j^	MH978179 ^j^	MH978187 ^j^	MH978182 ^j^	MH978185 ^j^
	GZUIFDX1	*Araneus* sp.	KU893152 ^j^	MH978178 ^j^	–	MH978181 ^j^	MH978184 ^j^
	GZUIFSN1	*Araneus* sp.	MH978177 ^j^	MH978180 ^j^	MH978188 ^j^	MH978183 ^j^	MH978186 ^j^
*Akanthomyces attenuatus*	CBS402.78	Leaf litter; * Acer saccharum*	AJ292434 ^f^	AF339565 ^f^	EF468782 ^f^	EF468888 ^f^	EF468935 ^f^
*Akanthomyces coccidioperitheciata*	NHJ6709	Araneae; spider	JN049865 ^f^	EU369042 ^f^	EU369025 ^f^	EU369067 ^f^	EU369086 ^f^
*Akanthomyces farinosa*	CBS541.81	–	–	AY6241807 ^b^	–	JQ4256867^b^	–
*Akanthomyces kanyawimiae*	TBRC7242	Araneae; spider	MF140751 ^i^	MF140718 ^i^	MF140838 ^i^	MF140784 ^i^	MF140808 ^i^
	TBRC7243	Unidentified	MF140750 ^i^	MF140717 ^i^	MF140837 ^i^	MF140783 ^i^	MF140807 ^i^
*Akanthomyces lecanii*	CBS101247	Hemiptera; *Coccus viridis*	JN049836 ^f^	AF339555 ^f^	DQ522359 ^f^	DQ522407 ^f^	DQ522466 ^f^
*Akanthomyces muscarius*	CBS470.73	–	–	MH878385 ^k^	–	–	–
	CBS455.70B	–	–	MH871560 ^k^	–	–	–
	CBS455.70C	–	–	MH871561 ^k^	–	–	–
*Akanthomyces noctuidarum*	BCC36265 T	Lepidoptera; Noctuidae	**MT356072**	**MT356084**	**MT477978**	**MT477994**	**MT477987**
	BBH16595		**MT356073**	**MT356085**	**MT477979**	**MT477995**	**MT478005**
	BCC47498		**MT356074**	**MT356086**	**MT477980**	**MT477996**	**MT477988**
	BCC28571		**MT356075**	**MT356087**	**MT477981**	**MT478009**	**MT478006**
*Akanthomyces pyralidarum*	BCC28816 T	Lepidoptera; Pyralidae	**MT356080**	**MT356091**	**MT477982**	**MT478000**	**MT478007**
	BCC32191		**MT356081**	**MT356092**	**MT477983**	**MT478001**	**MT477989**
	BCC40869		**MT356082**	**MT356093**	**MT477984**	**MT478002**	**MT477990**
	BCC29197		**MT356083**	**MT305694**	**MT508840**	**MT478003**	**MT477991**
*Akanthomyces sulphureus*	TBRC7248 T	Araneae; spider	MF140758 ^i^	MF140722 ^i^	MF140843 ^i^	MF140787 ^i^	MF140812 ^i^
	TBRC7249	Araneae; spider	MF140757 ^i^	MF140721 ^i^	MF140842 ^i^	MF140786 ^i^	MF140734 ^i^
*Akanthomyces thailandicus*	TBRC7245 T	Araneae; spider	MF140754 ^i^	–	MF140839 ^i^	–	MF140809 ^i^
*Akanthomyces tortricidarum*	BCC72638 T	Lepidoptera; Tortricidae	**MT356076**	**MT356088**	**MT478004**	**MT477997**	**MT477992**
	BCC41868		**MT356077**	**MT356089**	**MT477985**	**MT477998**	**MT478008**
	BCC28583		**MT356079**	**MT356090**	**MT477986**	**MT477999**	**MT477993**
*Akanthomyces tuberculatus*	HUA186131	Lepidoptera (adult moth)	–	MF416521 ^h^	MF416466 ^h^	–	–
*Akanthomyces waltergamsii*	TBRC7250	Araneae; spider	MF140749 ^i^	MF140715 ^i^	MF140835 ^i^	–	–
	TBRC7251	Araneae; spider	MF140747 ^i^	MF140713 ^i^	MF140833 ^i^	MF140781 ^i^	MF140805 ^i^
*Ascopolyporus polychrous*	PC 546	Plant	–	DQ118737 ^a^	DQ118745 ^a^	DQ127236 ^a^	–
*Ascopolyporus villosus*	ARSEF6355	Plant	–	AY886544 ^a^	DQ118750 ^a^	DQ127241 ^a^	–
*Beauveria acridophilla*	HUA179221	–	–	JQ895537 ^g^	JQ958615 ^g^	JX003853 ^g^	JX003843 ^g^
	MCA1181	Romaleidae; *Tropidacris cristata*	JQ958607 ^g^	JQ895542 ^g^	–	JX003856 ^g^	–
*Beauveria bassiana*	ARSEF1564	Lepidoptera; Arctiidae	HQ880761 ^e^	–	HQ880974 ^e^	HQ880833 ^e^	HQ880905 ^e^
*Beauveria blattidicola*	MCA1727	–	–	MF416539 ^h^	MF416483 ^h^	MF416640 ^h^	–
	MCA1814	–	–	MF416540 ^h^	MF416484 ^h^	MF416641 ^h^	–
*Beauveria brongniartii*	BCC16585	Coleoptera; *Anomala cuprea* (larva)	JN049867 ^f^	JF415967 ^f^	JF4160092 ^f^	JN049885 ^f^	JF415991 ^f^
	ARSEF617	Coleoptera; Scarabaeidae	HQ880782 ^e^	–	HQ880991 ^e^	HQ880854 ^e^	HQ880926 ^e^
*Beauveria caledonica*	ARSEF2567	Soil	HQ880817 ^e^	AF339520 ^g^	EF469057 ^g^	EF469086 ^g^	HQ880961 ^g^
*Beauveria malawiensis*	ARSEF7760	Coleoptera; Cerambycidae	–	–	DQ376246 ^g^	HQ880897 ^g^	HQ880969 ^g^
*Beauveria pseudobassiana*	ARSEF3405	Lepidoptera: Tortricidae	AY532022 ^e^	–	AY531931 ^e^	HQ880864 ^e^	HQ880936 ^e^
*Blackwellomyces cardinalis*	OSC93609	Lepidoptera; Tineidae (larva)	–	AY184962 ^g^	DQ522325 ^g^	DQ522370 ^g^	DQ522422 ^g^
	OSC93610		JN049843 ^f^	AY184963 ^f^	EF469059 ^f^	EF469088 ^f^	EF469106 ^f^
*Cordyceps amoenerosea*	CBS107.73	Coleoptera (pupa)	AY624168 ^b^	MG665224 ^i^	–	–	MG665234 ^i^
	CBS729.73	Coleoptera; Nitidulidae	AY624169 ^b^	MG665225 ^i^	HM161732 ^i^	–	MG665235 ^i^
*Cordyceps bifusispora*	spat08.129	–	–	MF416523 ^h^	MF416468 ^h^	MF416630 ^h^	–
	spat08.133.1	–	–	MF416524 ^h^	MF416469 ^h^	MF416631 ^h^	MF416434 ^h^
*Cordyceps blackwelliae*	TBRC7253	Lepidoptera	MF140739 ^i^	MF140705 ^i^	MF140825 ^i^	MF140774 ^i^	MF140798 ^i^
	TBRC7254	Lepidoptera	MF140738 ^i^	MF140704 ^i^	MF140824 ^i^	MF140773 ^i^	MF140797 ^i^
	TBRC7255	Lepidoptera	MF140737 ^i^	MF140703 ^i^	MF140823 ^i^	MF140772 ^i^	MF140796 ^i^
*Cordyceps caloceroides*	MCA2249	–	–	MF416525 ^h^	MF416470 ^h^	MF416632 ^h^	–
	QCNE186715	–	–	MF416526 ^h^	–	–	–
*Cordyceps cateniannulata*	TBRC7258	Araneae; spider	MF140753 ^i^	MF140729 ^i^	MF140850 ^i^	MF140767 ^i^	–
*Cordyceps coleopterorum*	CBS110.73	Coleoptera (larva)	AY624177 ^f^	JF415988 ^f^	JF416028 ^f^	JN049903 ^f^	JF416006 ^f^
*Cordyceps farinosa*	CBS111113	–	AY624181 ^b^	FJ765253 ^i^	GQ250022 ^i^	–	GU979973 ^i^
*Cordyceps fumosorosea*	CBS375.70	Food	AY624183 ^b^	MG665229 ^i^	HM161736 ^i^	–	MG665238 ^i^
	CBS107.10	–	AY624184 ^b^	MG665227 ^i^	HM161735 ^i^	–	MG665237 ^i^
*Cordyceps javanica*	TBRC7259	Lepidoptera	MF140745 ^i^	MF140711 ^i^	MF140831 ^i^	MF140780 ^i^	MF140804 ^i^
	TBRC7260	Lepidoptera	MF140744 ^i^	MF140710 ^i^	MF140830 ^i^	MF140779 ^i^	MF140803 ^i^
*Cordyceps kyusyuensis*	EFCC5886	Lepidoptera (pupa)	–	EF468813 ^c^	EF468754 ^c^	EF468863 ^c^	EF468917 ^c^
*Cordyceps lepidopterorum*	TBRC7263	Lepidoptera (larva)	MF140765 ^i^	MF140699 ^i^	MF140819 ^i^	MF140768 ^i^	MF140792 ^i^
	TBRC7264		MF140766 ^i^	MF140700 ^i^	MF140820 ^i^	MF140769 ^i^	MF140793 ^i^
*Cordyceps militaris*	OSC93623	Lepidoptera (pupa)	–	EF468821 ^h^	EF468762 ^h^	EF468869 ^h^	–
*Cordyceps ochraceostromata*	ARSEF5691	Lepidoptera	–	EF468819 ^h^	EF468759 ^h^	EF468867 ^h^	EF468921 ^h^
*Cordyceps ninchukispora*	spat08.115	–	–	MF416532 ^h^	MF416476 ^h^	MF416635 ^h^	MF416439 ^h^
	spat09.021	–	–	MF416533 ^h^	MF416477 ^h^	MF416636 ^h^	–
*Cordyceps rosea*	spat09.053	–	–	MF416536 ^h^	MF416480 ^h^	MF416637 ^h^	MF416442 ^h^
*Cordyceps takaomontana*	BCC12688	Lepidoptera	EU807996 ^i^	–	–	–	–
*Cordyceps tenuipes*	TBRC7265	Lepidoptera (pupa)	MF140741 ^i^	MF140707 ^i^	MF140827 ^i^	MF140776 ^i^	MF140800 ^i^
	TBRC7266		MF140742 ^i^	MF140708 ^i^	MF140828 ^i^	MF140777 ^i^	MF140801 ^i^
*Engyodontium aranearum*	CBS309.85	Araneae; spider	–	AF339526 ^c^	DQ522341 ^c^	DQ522387 ^c^	DQ522439 ^c^
*Gibellula pulchra*	NHJ10808	Araneae; spider	–	EU369035 ^d^	EU369018 ^d^	EU369056 ^d^	EU369076 ^d^
*Gibellula ratticaudata*	ARSEF1915	Araneae; spider	–	DQ518777 ^d^	DQ522360 ^d^	DQ522408 ^d^	DQ522467 ^d^
*Gibellula* sp.	NHJ5401	Araneae; spider	–	–	–	EU369059 ^d^	EU369097 ^d^
*Hevansia arachnophila*	NHJ10469	Araneae; spider	–	EU369031 ^d^	EU369008 ^d^	EU369047 ^d^	–
*Hevansia cinerea*	NHJ3510	Araneae; spider	–	–	EU369009 ^d^	EU369048 ^d^	EU369070 ^d^
*Hevansia nelumboides*	BCC2093	–	–	MF416530 ^h^	MF416473 ^h^	–	MF416437 ^h^
*Hevansia novoguineensis*	NHJ11923	Araneae; spider	–	EU369032 ^d^	EU369013 ^d^	EU369052 ^d^	EU369072 ^d^
	NHJ13161	Araneae; spider	–	–	EU369011 ^d^	EU369050 ^d^	–
*Hevansia websteri*	BCC23860	–	–	–	GQ250030 ^l^	–	–
*Isaria farinosa*	OSC111005	–	–	DQ518772 ^h^	DQ522348 ^h^	DQ522394 ^h^	–
	OSC111006	–	–	EF469080 ^h^	EF469065 ^h^	EF469094 ^h^	–
*Isaria* sp.	spat09.050	–	–	MF416559 ^h^	MF416506 ^h^	MF416663 ^h^	MF416457 ^h^
	spat09.051	–	–	MF416560 ^h^	MF416507 ^h^	MF416664 ^h^	MF416458 ^h^
*Lecanicillium antillanum*	CBS350.85 T	Fungi; agaric (Hymenomycetes)	–	AF339536 ^d^	DQ22350 ^d^	DQ522396 ^d^	DQ522450 ^d^
*Lecanicillium psalliotae*	CBS101270	Soil	–	EF469081 ^c^	EF469066 ^c^	EF469095 ^c^	EF469113 ^c^
	CBS532.81		–	AF339560 ^c^	EF469067 ^c^	EF469096 ^c^	EF469112 ^c^
*Purpureocillium lilacinum*	CBS284.36	Soil	AY624189 ^b^	FR775484 ^c^	EF468792 ^c^	EF468898 ^c^	EF468941 ^c^
	CBS431.87	Nematoda; *Meloidogyne* sp.	AY624188 ^f^	EF468844 ^f^	EF468791 ^f^	EF468897 ^f^	EF468940 ^f^
*Samsoniella aurantia*	TBRC7271	Lepidoptera	MF140764 ^i^	MF140728 ^i^	MF140846 ^i^	MF140791 ^i^	MF140818 ^i^
	TBRC7272		MF140763 ^i^	MF140727 ^i^	MF140845 ^i^	–	MF140817 ^i^
*Samsoniella inthanonensis*	TBRC7915	Lepidoptera (pupa)	MF140761 ^i^	MF140725 ^i^	MF140849 ^i^	MF140790 ^i^	MF140815 ^i^
	TBRC7916		MF140760 ^i^	MF140724 ^i^	MF140848 ^i^	MF140789 ^i^	MF140814 ^i^
*Simplicillium lamellicola*	CBS116.25	Soil	AJ292393 ^f^	AF339552 ^f^	DQ522356 ^f^	DQ522404 ^f^	DQ522462 ^f^
*Simplicillium lanosoniveum*	CBS704.86	Fungi; *Hemileia vastatrix*	–	AF339553 ^c^	DQ522358 ^c^	DQ522406 ^c^	DQ522464 ^c^
	CBS101267		AJ292395 ^f^	AF339554 ^f^	DQ522357 ^f^	DQ522405 ^f^	DQ522463 ^f^
*Simplicillium obclavatum*	CBS311.74	Air above sugarcane field	–	AF339517 ^c^	EF468798 ^c^	–	–
*Torrubiella wallacei*	CBS101237	Lepidoptera	–	AY184967 ^c^	EF469073 ^c^	EF469102 ^c^	EF469119 ^c^
*Verticillium* sp.	CBS102184	–	–	AF339564 ^h^	EF468803 ^h^	EF468907 ^h^	EF468948 ^h^

Note. The accession numbers in bold font refer to sequences generated in this study. Strain numbers with T are type species. References. ^a^Chaverri et al. (2005), ^b^Luangsa-ard et al. (2005), ^c^Sung et al. (2007), ^d^Johnson et al. (2009), ^e^Rehner et al. (2011), ^f^Kepler et al. (2012), ^g^Sanjuan et al. (2014), ^h^Kepler et al. (2017), ^i^Mongkolsamrit et al. (2018), ^j^Chen et al. (2018), ^k^Kuephadungphan et al. (2018), ^l^Vu et al. (2019).

**Figure 1.**
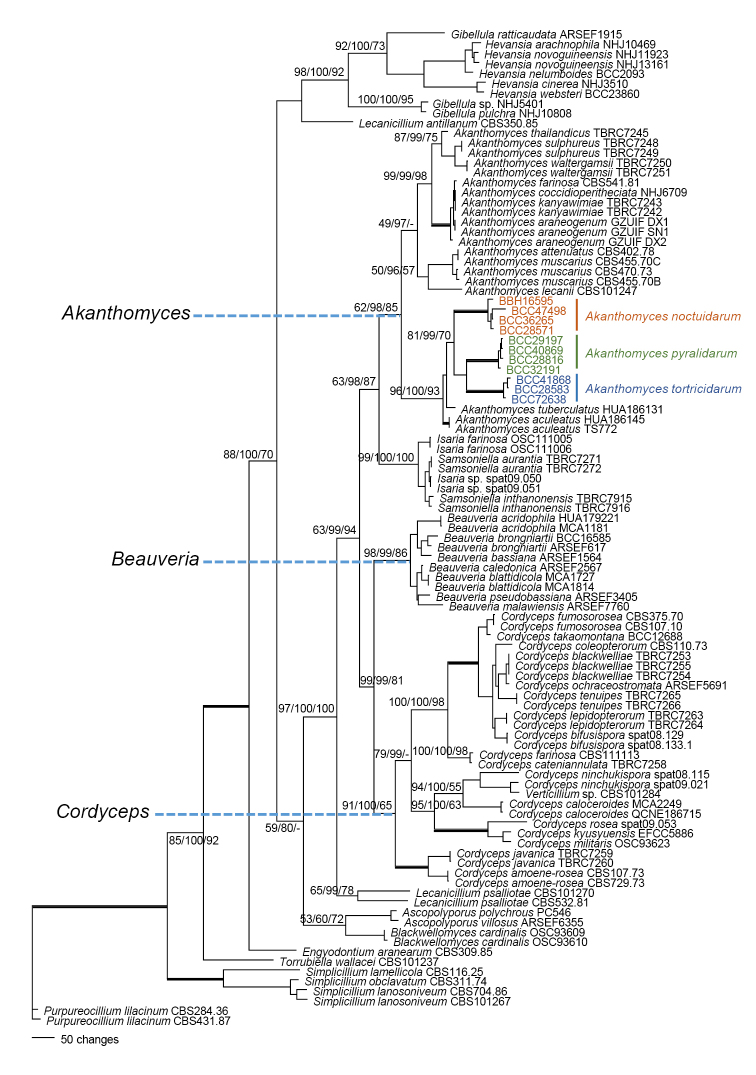
Phylogenetic tree based on combined dataset of LSU, *TEF*, *RPB1* and *RPB2*, sequences showing the relationship of *Akanthomyces* from Thailand with other species of Cordycipitaceae. Numbers above lines at significant nodes represent Maximum likelihood bootstrap values, Bayesian posterior probabilities, and MP bootstrap values. Bold lines mean support for the three analyses were 100%.

The phylogenetic analyses were run using a combined dataset comprising four loci: LSU, *TEF*, *RPB1*, and *RPB2*. The combined dataset included 3511 characters, of which 2053 characters were constant, 231 were parsimony-uninformative, and 1227 were parsimony-informative. Gaps were treated as missing data. The maximum parsimony analyses resulted in 31 equally most parsimonious trees, of which one is shown in Figure 1 (tree length = 6438 steps; consistency index [CI] = 0.3458; retention index [RI] = 0.7410; homoplasy index [HI] = 0.6542). The result of MrModeltest selected the general time-reversible (GTR) model with proportion in invariable sites (I) and gamma distribution (G) (GTR+I +G) (Lanave et al. 1984) as the best-fit model by the Akaike Information Criterion (AIC) in MrModeltest 2.2. The parameters included base frequencies A = 0.4768, C = 0.7426, G = 1.000, T = 1.2195 and the rate matrix for the substitution model: [A–C] = 0.2693, [A–G] = 0.2507, [A–T] = 0.2694, [C–G] = 0.2159, [C–T] = 1.1151, [G–T] = 1.000. For among-site variation, the proportion of invariable sites (I) was 0.3370 and the gamma distribution shape parameter (G) was 0.5036. This model was used in MrBayes and RAxML. MP and RAxML trees are provided as Suppl. materials 1, 2.

## Taxonomy

### Akanthomyces noctuidarum

Taxon classificationFungiHypocrealesCordycipitaceae

Aini, Luangsa-ard, Mongkolsamrit & Thanakitpipattana sp. nov.

03ED234C-1C2E-5BD0-9146-DD13D4C2C0F1

835652

Figure 2

#### Type.

Thailand. Narathiwat Province, Hala Bala Wildlife Sanctuary, Headquarter Nature Trail; 5°928'N, 101°883'E; on adult moth; 3 Mar 2009; K. Tasanathai (KT), P. Puyngain (PP), T. Chohmee (TC) (holotype BBH 26019 dried culture; ex-type living culture BCC 36265). GenBank: ITS = MT356072, LSU = MT356084, *TEF* = MT477978, *RPB1* = MT477994, *RPB2* = MT477987.

#### Etymology.

Referring to the host (Noctuidae, Lepidoptera) where the fungus was found.

#### Description.

Teleomorph: Adult moth attached to the midrib of monocotyledonous leaf or undersides of dicotyledonous leaf covered by white to cream mycelium (OAC816). Stroma arising from host body and wing veins, white to cream, cylindrical, length ca. 5 mm. Perithecia superficial, orange to light brown (OAC825), few to numerous, crowded at the tip of the stroma or growing directly from mycelium in host body and wing veins, ovoid, (530–)623–993(–1000) × (290–)308–413(–425) µm. *Asci* cylindrical, hyaline, (170–)196–423(–550) × (2–)2.7–3.8(–4) µm. Ascospores cylindrical, filiform, hyaline, multi-septate, breaking into one-celled fragments at maturity, (6–)7–10.7(–13) × 1 µm.

Anamorph: Synnemata arising from moth body and wing veins, white to cream (OAC816), erect, simple, cylindrical to clavate, (650–)668–1191(–1500) × (50–)53.4–102(–120) µm. Conidiogenous cells produced along the synnemata, monophialidic or polyphialidic. Phialides cylindrical with papillate end, hyaline, (5–)6.8–9(–10) × (1.8–) 2–2.4(–3) µm. Conidia cylindrical with round end, hyaline, (3–)3.5–4.5(–6) × 1 µm.

#### Culture characters.

Colony on PDA growing with a diameter of 20–24 mm in 14 days, circular, flat to raised, entire edges, white (OAC909) and fluffy mycelium. Colony reverse cream (OAC814). Colony on OA growing with a diameter of 20–25 mm in 14 days, circular, flat to raised, entire, white (OAC 909) and fluffy mycelium. Colony reverse uncolored. Conidia and reproductive structures not observed on both, PDA and OA in 14 days.

**Figure 2.**
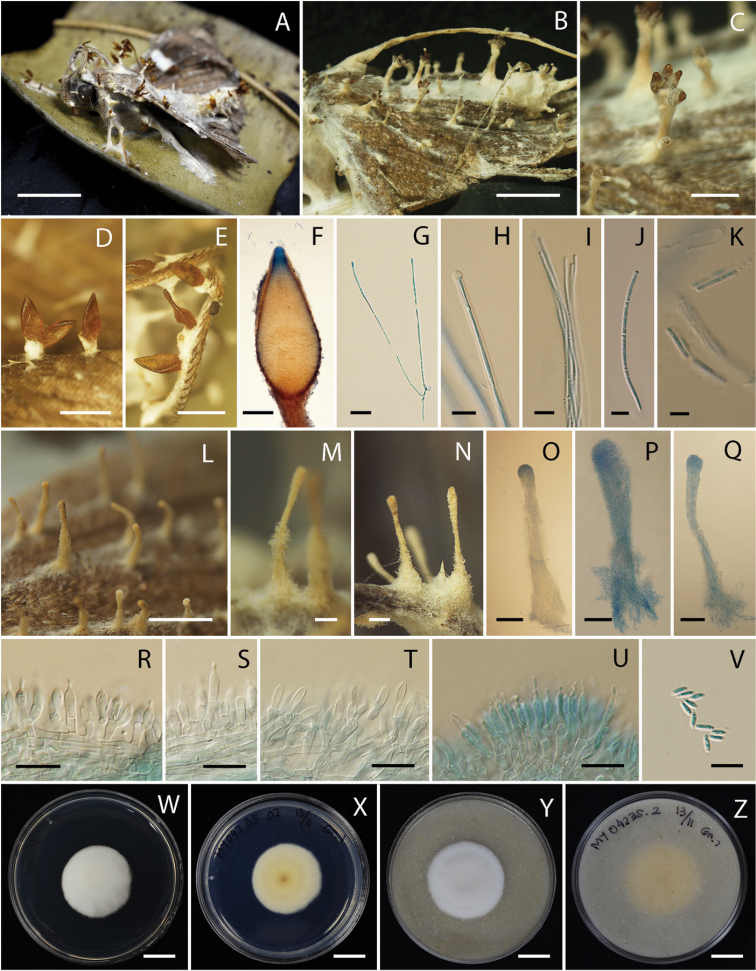
*Akanthomyces noctuidarum* (BBH 26019, BCC 36265) **A, B** fungus on adult moth **C–F** perithecia **G** asci **H** tip of ascus **I** ascus with ascospores **J** ascospores with clear septae **K** ascospores break into part-spores **L–Q** synnemata **R–T** phialides through the length of synnema **U** phialides at the tip of synnema **V** conidia **W, X** culture on PDA 14 days **X** reverse **Y, Z** culture on OA 14 days **Z** reverse. Scale bars: 1 cm (**A, B, W, X, Y, Z**); 5 mm (**C, I, J, K**); 1 mm (**D, E, L**); 200 µm (**F, M, N, O, P, Q**); 50 µm (**G**); 10 µm (**H, R, S, T, U, V**).

#### Distribution.

Thailand, known from various national parks throughout the country.

#### Ecology.

All specimens were found on the underside of leaves of plants.

#### Additional specimens examined.

Thailand. Nakhon Ratchasima Province, Khao Yai National Park, Km.29; 14°711'N, 101°421'E; on adult moth; 24 Jan 2006; KT, W. Chaygate (WC), S. Sivichai, Le Tan Hung (BBH16595). Narathiwat Province, Hala Bala Wildlife Sanctuary, Headquarter Nature Trail; 5°928'N, 101°883'E; on adult moth; 19 Feb 2011; KT (BBH30267, BCC 47498). Kamphaeng Phet Province, Khlong Lan National Park, Saphan Ton Nature Trail; 16°203'N, 99°321'E; on adult moth; 6 Nov 2007; BT, KT, WC, S. Mongkolsamrit (SM), P. Srikitikulchai (PS), R. Ridkaew (RR), A. Khonsanit (AK) (BBH22738, BCC 28571).

#### Notes.

This species produced both, anamorph and teleomorph. The type strain of this species, BBH 26019/ BCC 36265, consisted of both, anamorph and teleomorph. The other strains produced only one morph on the insect, either anamorph or teleomorph.

### Akanthomyces pyralidarum

Taxon classificationFungiHypocrealesCordycipitaceae

Aini, Luangsa-ard, Mongkolsamrit & Thanakitpipattana sp. nov.

4A4A3532-007C-5DD0-9120-2E31BE126704

835653

Figure 3

#### Type.

Thailand. Kanchanaburi Province, Thung Yai Naresuan Wildlife Sanctuary, Krathon Ruesi Nature Trail; 14°746'N, 98°625'E; on adult moth; 11 Dec 2007; KT, SM, RR, B. Thongnuch (BT) (holotype BBH23823 dried culture, ex-type living culture BCC 28816). GenBank: ITS = MT356080, LSU = MT356091, *TEF* = MT477982, *RPB1* = MT478000, *RPB2* = MT478007.

#### Etymology.

Refers to the host (Pyralidae, Lepidoptera) of the fungus.

#### Description.

Teleomorph: Adult moth attached on the undersides of dicotyledonous leaf covered by white to cream mycelium (OAC816). Stroma arising from host body and wings, white to cream (OAC816), cylindrical. Perithecia superficial, crowded at the tip of stroma or growing directly from mycelium that covers the host body, few to numerous, ovoid to obpyriform, (290–)342–580(–650) × (150–)186–291(–340) µm. Asci cylindrical, the bottom of asci thicker than the middle part, (170–)222–329(–360) × (2–)2.5–3.3(–4) µm. Ascospores hyaline, filiform, multi-septate, discharged into part-spores, (5–)5.9–9.4(–12) × 1 µm.

#### Culture characters.

Colonies on PDA growing with a diameter of 23–28 mm in 14 days, white (OAC909), circular, flat, entire. Colony reverse pale yellow (OAC856) at the center. Conidia and reproductive structures not observed. Colonies on OA growing with a diameter of 27–30 mm in 14 days, white (OAC909), circular, flat, entire. Colony reverse uncolored. Conidia and reproductive structures not observed.

**Figure 3.**
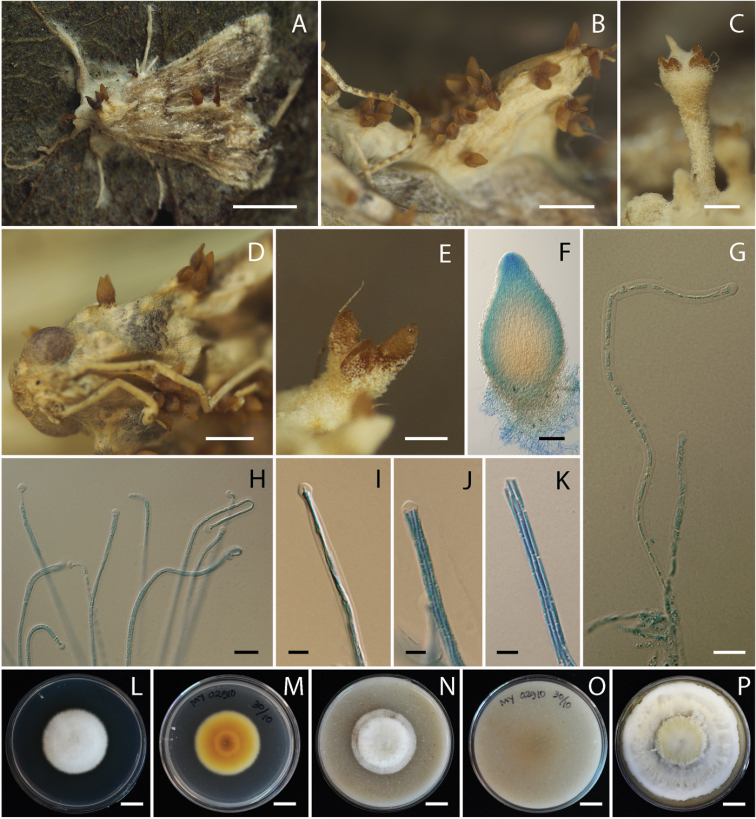
*Akanthomyces pyralidarum* (BBH 23823, BCC 28816) **A** fungus on adult moth **B–F** perithecia **G, H** asci **I** tip of ascus with immature ascospore **J** tip of ascus with mature ascospores **K** ascospores **L, M** culture on PDA 14 days **M** reverse **N, O** culture on OA 14 days **O** reverse **P** culture on OA 28 days. Scale bars: 1 cm (**A, L, M, N, O, P**); 1 mm (**B**); 500 µm (**C, D, E**); 100 µm (**F**); 10 µm (**G, H**); 5 µm (**I, J, K**).

#### Distribution.

Thailand, known from various national parks throughout the country.

#### Ecology.

All specimens are found on the underside of leaves of plants.

#### Additional specimens examined.

Thailand. Chiang Mai Province, Huai Nam Dang National Park, Pong Dueat Pa Pae Geyser; 19°121'N, 98°943'E; on adult moth; 5 Sep 2008; KT, WC, PS, AK, SM (BBH 24623, BCC 32191). Phetchabun Province, Nam Nao National Park, Headquarter Nature Trail; 16°768'N, 101°671'E; on adult moth; 24 Nov 2009; KT, TC, AK (BBH 27293, BCC 40869). Kanchanaburi Province, Thung Yai Naresuan Wildlife Sanctuary, Thi Khong Protect Forest Unit; 14°746'N, 98°625'E; on adult moth; 12 Dec 2007; KT, SM, RR, BT (BBH 23778, BCC 29197).

#### Notes.

*Akanthomyces pyralidarum* is found only in its teleomorph state. This species differs from *Akanthomyces noctuidarum* by having smaller perithecia (290–650 × 150–340 µm) than *A. noctuidarum* (530–1000 × 290–425 µm).

### Akanthomyces tortricidarum

Taxon classificationFungiHypocrealesCordycipitaceae

Aini, Luangsa-ard, Mongkolsamrit & Thanakitpipattana sp. nov.

44263CF5-64D3-5F00-AE8C-F350554C70F3

835654

Figure 4

#### Type.

Thailand. Nakhon Ratchasima Province, Khao Yai National Park, Mo Sing To Nature Trail; 14°711'N, 101°421'E; on adult moth; 6 Jun 2014; W. Noisripoom, PS, TC, S. Sommai, R. Somnuk (holotype BBH 38669 dried culture, ex-type living culture BCC 72638). GenBank: ITS = MT356076, LSU = MT356088, *TEF* = MT478004, *RPB1* = MT477997, *RPB2* = MT477992.

#### Etymology.

Refers to the host (Tortricidae, Lepidoptera) of the fungus.

#### Description.

Anamorph: Specimens examined in this study can be found on the underside of dicotyledonous leaves and palm leaf. The hosts were adult moths, ca. 4–9 × 1–2 mm. Two types of synnemata were produced on insect hosts. Several long synnemata arose at the head and in the middle of the host body, white to cream, up to 5 mm long and ca. 120–150 µm wide, rarely branched, cylindrical to clavate with acute or blunt end. Conidiogenous cells produced along synnemata, monophialidic or polyphialidic. Phialides (5–)6–8(–10) × (1.8–)2–2.7(–3) μm, cylindrical to ellipsoidal with papillate end. Conidia smooth-walled, hyaline, single-celled, fusoid, (2–)2.5–3(–3.2) × (0.8–)1–1.4(–2) µm. Several short synnemata arose on moth body, wings, and legs, white to cream, (197–)200–267(–300) × (15–)17.7–31.6(–40) µm, with diameter of the tip (43–)51.5–73(–75) µm, cylindrical with subglobose or oblong end. Conidiogenous cells produced at the end of synnemata, monophialidic or polyphialidic. Phialides (5–)6.2–8.3(–10) × (1.8–)2–2.5(–3) μm, cylindrical to ellipsoidal with papillate end. Conidia smooth-walled, hyaline, single-celled, fusoid, (1–)1.8–2.7(–3) × 1–2 µm. Phialides and conidia from both long and short synnemata were on the same size range.

#### Culture characters.

Colonies on PDA growing with a diameter of 25–31 mm in 14 days, white (OAC909), circular, flat, entire, reverse pale yellow (OAC858). Mycelium smooth, septate, hyaline. Colonies on OA growing with a diameter of 18–25 mm in 14 days, circular, flat, entire, white (OAC909), reverse brownish yellow (OAC812). Mycelium smooth, septate, hyaline. Conidia and reproductive structures not produced on both, PDA and OA in 14 days.

**Figure 4.**
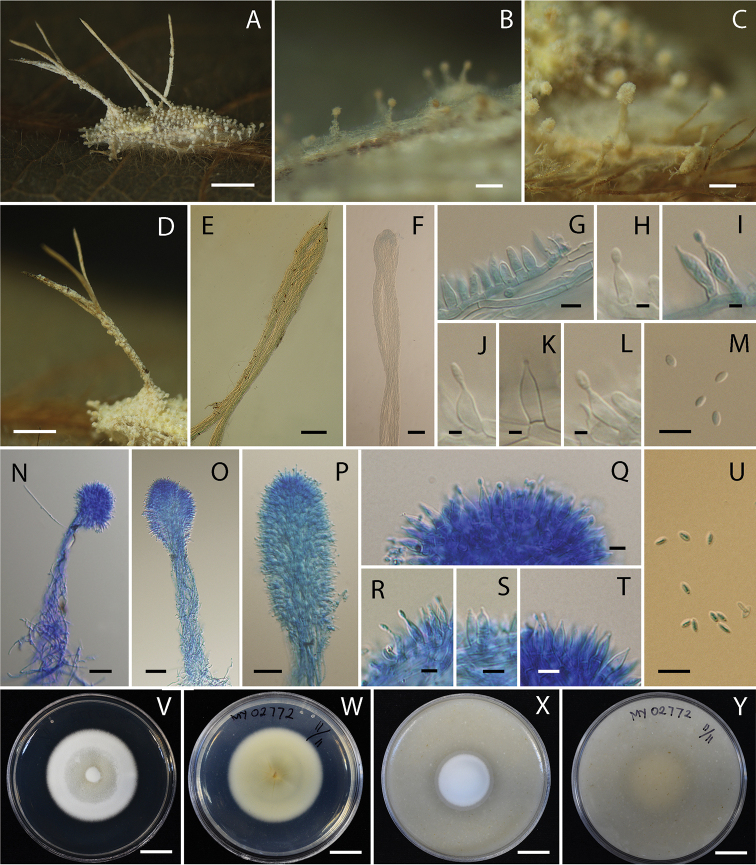
*Akanthomyces tortricidarum* (BBH 38669, BCC 72638) **A** fungus on adult moth **B, C, N–P** short synnemata **D–F** long synnemata **G–L** phialides from long synnema **M** conidia from long synnema **Q–T** phialides from short synnema **U** conidia from short synnema **V, W** culture on PDA 14 days **W** reverse **X, Y** culture on OA 14 days **Y** reverse. Scale bars: 2 mm (**A**); 200 µm (**B, E**); 100 µm (**C, F**); 500 µm (**D**); 5 µm (**G, M, Q, R, S, T, U**); 2 µm (**H, I, J, K, L**); 30 µm (**N, O, P**); 1 cm (**V, W, X, Y**).

#### Distribution.

Thailand, known from various national parks throughout the country.

#### Ecology.

All specimens are found on the underside of leaves of plants.

#### Additional specimens examined.

Thailand. Nakhon Ratchasima Province, Khao Yai National Park, Mo Sing to Nature Trail; 14°711'N, 101°421'E; on adult moth; 7 Apr 2010; KT, SM, TC, AA, RR (BBH 28530, BCC 41868). Nakhon Ratchasima Province, Khao Yai National Park, Mo Sing to Nature Trail; 14°711'N, 101°421'E; on adult moth; 11 Nov 2009; KT, SM, TC, RR, M. Sudhadham, AK (BBH 27283, BCC 40005). Kamphaeng Phet Province, Khlong Lan National Park, Saphan Ton Nature Trail; 16°203'N, 99°321'E; on adult moth; 6 Nov 2007; KT, SM, PS, BT, RR, AK, WC (BBH 23097, BCC 28583).

#### Notes.

*Akanthomyces tortricidarum* is found only in its anamorph state. This species differs from *A. noctuidarum* by having smaller conidia (2–3 × 1 µm) than *A. noctuidarum* (3–6 × 1 µm). Furthermore, the shape of conidia of *A. tortricidarum* is fusoid, while conidia of *A. noctuidarum* is cylindrical with a round end.

## Discussion

The genus *Akanthomyces* established by Lebert (1858) was revised by Mains (1950). This genus is characterized by cylindrical synnemata covered by a hymenium-like layer of phialides producing single-celled catenulate conidia (Samson 1974). Presently, 20 *Akanthomyces* species have been formally described (Kepler et al. 2017; Mongkolsamrit et al. 2018), while eight species of *Akanthomyces* on spiders were transferred to the genus *Hevansia*. *Hevansia* includes the type species *Hevansia novoguineensis* (previously described as *Akanthomyces novoguineensis*), which differs from *Akanthomyces* by the immersed perithecia in a disk sitting at the top of a well-formed stipe. However, now it has to be an akanthomyces-like teleomorph (Kepler et al. 2017). *Akanthomyces* is considered as a synonym of *Lecanicillium*, an anamorph within Cordycipitaceae with verticillium-like morphologies (Gams and Zare 2001). *Lecanicillium* does not form a single monophyletic clade and species within this genus are distributed throughout Cordycipitaceae (Sukarno et al. 2009). Based on the molecular analyses from five nuclear genes (SSU, LSU, *TEF*, *RPB1*, and *RPB2*), Kepler et al. (2017) proposed that *Lecanicillium* should be rejected and *Akanthomyces* has priority by date over this genus. The type species of *Lecanicillium*, *L. lecanii* as well as some other species (*L. attenuatum*, *L. muscarium*, and *L. sabanense*) have phylogenetic affinities to *Akanthomyces* (Chiriví-Salomón et al. 2015)

The type species of *Akanthomyces*, *A. aculeatus* and another *Akanthomyces* species on moth, *A. pistillariiformis* (= *A. tuberculatus*), were the closest related species to the three new species described here. Two of three new species were found in their anamorph state. Fortunately, in *A. noctuidarum* both, teleomorph and anamorph are present in the same specimen. The anamorph comparison between some species within *Akanthomyces* is shown in Table 2. The conidia of *A. noctuidarum* and *A. aculeatus* are almost in the same size (*A. noctuidarum*; 3–6 × 1 µm, *A. aculeatus*; 3–6 × 2–3 µm). However, the conidial shape of *A. noctuidarum* is cylindrical with a round end while *A. aculeatus* is ellipsoid or obovoid. *Akanthomyces noctuidarum* has the smallest synnemata compared to all the others (*A. noctuidarum*; 650–1500 µm, *A. aculeatus*; 1–8 × 0.1–0.5 mm, *A. tuberculatus*; 1–6 mm × 50–300 µm). *Akanthomyces noctuidarum* also has smaller phialides than both aforementioned species (5–10 × 2–3 µm, *A. aculeatus*; 6–16 × 2.5–4 µm, *A. tuberculatus*; 7–10.5 × 2.7–3.5 µm) with cylindrical shape and papillate at the end.

**Table 2.** Morphological comparisons between anamorph of closely related *Akanthomyces* species used in this study.

Species	Host	Synnemata	Phialides	Conidia
*Akanthomyces aculeatus* ^2^	Moth (Lepidoptera)	Yellowish, cylindrical, narrowing upward, 1–8 mm long and 0.1–0.5 mm wide	Subcylindric to narrowly ellipsoidal, 6–16 × 2.5–4 µm	Ellipsoidal or obovoid, 3–6 × 2–3 µm
*Akanthomyces angustispora* ^2^	Coleoptera larva	Flesh colored, simple or branched, 8–13 mm long and 0.2–0.6 mm wide	Oblong or narrowly ellipsoidal, 6–14 × 3–4 µm	Narrowly clavate, 4.5–6 × 1.2–1.4 µm
*Akanthomyces arachnophilus* ^4^	Spider (Araneae)	Creamish yellow to pale brown, simple or branched, cylindrical, 2.5–5 mm × 50–75 µm	Globose, 3.2–4.3 × 6.5–8.5 µm	Fusiform, 4.5–5.5(–6) × 1.5–3 µm
*Akanthomyces araneogenum* ^5^	Spider	Conidiophores mononematous or synnematous, 21.6–48 × 1.2–2.2 μm, penicillium-like from hyphae directly	Cylindrical, somewhat inflated base, tapering to a thin neck, 4.3–17.3 × 0.9–3.1 μm	Globose, 1.3–2.4 μm in diam, or ellipsoidal, 2.1–3.3 × 1.1–1.6 μm
*Akanthomyces gracilis* ^4^	Hymenoptera, Coleoptera, Lepidoptera (moth larvae) Heteroptera, Homoptera	White to yellow-brown, simple, rarely branched, cylindrical, usually 0.7–2 mm × 100–400 µm, occasionally up to 30 mm long and 0.5 mm wide	Cylindrical, 7–10 × 1.5–2.5 µm	Ellipsoidal, fusiform, 2.5–3 × 1–1.6 µm
*Akanthomyces kanyawimiae* ^3^	Spider (Araneae)	Up to 1.5 mm long, up to 400 µm wide; loosely covered by dense white to cream mycelia	Cylindrical to ellipsoidal, (7–)8–10.5(–12) × 2–3 µm	Fusiform or lemon-shaped, (2–)2.5–3.5(–4) × 1–2 µm
*Akanthomyces noctuidarum* ^1^	Lepidoptera; Noctuidae	White to cream (OAC816), simple, cylindrical to clavate, (650–)668–1191(–1500) × (50–)53–102(–120) µm	Cylindrical with papillate end, (5–)6.8–9(–10) × (1.8–)2–2.4(–3) µm	Cylindrical with round end, (3–)3.5–4.7(–6) × 1 µm
*Akanthomyces pistillariiformis*^4^ (= *A. tuberculatus*)	Moth (Lepidoptera)	White to creamish, simple, occasionally branched, cylindrical to clavate and stipitate, 1–6 mm long and 50–300 µm wide	Cylindrical, 7–10.5 × 2.7–3.5 µm	Cylindrical to narrowly fusiform, 4.5–6 × 1.2–1.5 µm
*Akanthomyces suphureus* ^3^	Spider (Araneae)	–	Cylindrical, (5–)7.5–11(–12) × 2–2.5 µm	Cylindrical to ellipsoidal, (3–)4(–5) × (1–)1.5(–2) µm
*Akanthomyces tortricidarum* ^1^	Lepidoptera; Tortricidae	**Long** synnemata white to cream, rarely branched, cylindrical to clavate with acute or blunt end, up to 5 mm long and wide ca. 120–150 µm.	Cylindrical to ellipsoidal with papillate end, (5–)6–8(–10) × (1.8–)2–2.7(–3) μm	Fusoid, (2–)2.5–3(–3.2) × 1–2 µm
		**Short** synnemata white to cream, cylindrical with subglobose or oblong at the end, (197–)200–267(–300) × (15–)17.7–31.6(–40) µm, with diameter of the tip (43–)51.5–73(–75) µm	Cylindrical to ellipsoidal with papillate end, (5–)6.2–8.3(–10) × (1.8–)2–2.5(–3) μm	Fusoid, (1–)1.8–2.7(–3) × 1–2 µm
*Akanthomyces waltergamsii* ^3^	Spider (Araneae)	White to cream synnemata up to 1.5 mm long and ca. 100–120 µm wide	Cylindrical to ellipsoidal, (7–)8.5–11(–12) × 2.5–3 µm	Ellipsoidal, fusiform, (3–)4–5.5(–6) × 1.5–2 µm

Notes. ^1^Current study, ^2^Mains (1950), ^3^Mongkolsamrit et al. (2018), ^4^Samson and Evans (1974), ^5^Chen et al. (2018).

*Akanthomyces tortricidarum* was distinguished from the others species by having two different types of synnemata. The long synnemata of *A. tortricidarum* are cylindrical to clavate with acute or blunt ends. The hyphae diverged in the upper portion of the synnema and repeatedly branched more or less dichotomously, whereas the phialides were terminal on the branches. At the lower portion of synnema, the phialides were produced either as lateral cells or frequently as terminal cells of short lateral branches produced along the entire length of the outer hyphae of the synnema. The production of phialides was abundant at the upper portion of the synnema, resulting in a compact hymenial layer, whereas the phialides at the lower portion of the synnema were scattered and well separated from each other. Unlike the long synnemata, the hymenium-like layer of phialides on the short synnemata was limited to its upper part and the lower portion was sterile, forming a stipe. In the upper portion of the short synnema, the hyphae diverged and repeatedly branched more or less dichotomously and terminated with phialides. However, at the lower portion, the outer longitudinal hyphae did not produce any lateral phialides or lateral branches bearing phialides. This character was similar to the genus *Insecticola* proposed by Mains (1950). However, Samson and Evans (1974) transferred all members of this genus to *Akanthomyces* because variations in these characters did not support the distinction. The shape of synnemata and arrangement of phialides from *A. noctuidarum* and long synnemata from *A. tortricidarum* were similar. Nevertheless, *A. tortricidarum* differs from *A. noctuidarum* by having smaller conidia (2–3 × 1 µm) than *A. noctuidarum* (3–6 × 1 µm). Furthermore, the shape of conidia in *A. tortricidarum* is fusoid, while the conidia of *A. noctuidarum* is cylindrical with rounded ends.

The teleomorph comparison between some species within *Akanthomyces* is shown in Table 3. *Akanthomyces noctuidarum* and *A. pyralidarum* differed from *A. tuberculatus* by the size of ascospores, asci, and perithecia. *Akanthomyces tuberculatus* has smaller ascospores measuring 2–6 × 0.5–1 µm, whereas *A. noctuidarum* and *A. pyralidarum* have larger ascospores at 6–13 × 1 µm and 5–12 × 1 µm, respectively. However, all three of them have the same shape of ascospore and asci. *Akanthomyces pyralidarum* has the smallest size of asci (*A. pyralidarum*; 170–360 × 2–4 µm, *A. noctuidarum*; 170–550 × 2–4 µm, and *A. tuberculatus*; 300–600 × 4–5 µm). The shape of perithecia from *A. noctuidarum* is ovoid, while *A. pyralidarum* is ovoid to obpyriform and *A. tuberculata* is narrowly ovoid or conoid. *Akanthomyes pyralidarum* also has the smallest perithecia compared to the other species in the genus (*A. pyralidarum*; 290–650 × 150–340 µm, *A. tuberculatus*; 420–900 × 180–370 µm, *A. sulphureus*; 650–680 × 240–330 µm, *A. thailandicus*; 700–850 × 300–400 µm, and *A. noctuidarum*; 530–1000 × 290–425 µm). Moreover, *A. sulphureus* and *A. thailandicus* are found on spiders (Araneae) while the others were found on moths.

All strains from these species did not produce conidia or reproductive structures when grown on PDA and OA for 14 days at 25 °C. Nevertheless, one strain from *A. pyralidarum* (BCC 29197) started to produce a synnemata-like structure on OA after 28 days. However, this synnemata-like structure was sterile and did not produce any phialides or conidia. Overall, fungal growth was faster in OA than in PDA.

**Table 3.** Morphological comparisons between teleomorph of closely related *Akanthomyces* species used in this study.

Species	Host	Perithecia	Asci	Ascospores
*Akanthomyces noctuidarum* ^1^	Lepidoptera; Noctuidae	Superficial, orange to light brown, ovoid, (530–)623–993(–1000) × (290–)308–413(–425) µm	Cylindrical, (170–)196–423(–550) × (2–)2.7–3.8(–4) µm	Cylindrical, filiform, multi-septate, part-spores, (6–)7–10.7(–13) × 1 µm
*Akanthomyces pyralidarum* ^1^	Lepidoptera; Pyralidae	Superficial, ovoid to obpyriform, (290–)342–580(–650) × (150–)186–291(–340) µm	Cylindrical, (170–)222–329(–360) × (2–)2.5–3.3(–4) µm	Filiform, multi-septate, part-spores, (5–)5.9–9.4(–12) × 1 µm
*Akanthomyces suphureus* ^2^	Spider (Araneae)	Superficial, ovoid, (650–)676(–680) × (240–)324.5(–330) µm	Cylindrical, up to 500 µm long, 2–3 µm wide	Whole, filiform, (300–) 336(–450) × 1–1.5 µm
*Akanthomyces thailandicus* ^2^	Spider (Araneae)	Superficial, narrowly ovoid, (700–)752–838(–850) × (300–)305–375(–400) µm	Cylindrical, up to 550 µm long, 5–7 µm wide	Cylindrical, multi-septate, part-spores, 4–6 × 1–1.5 µm
*Akanthomyces tuberculatus^3^* (= *C. tuberculata*)	Moth (Lepidoptera)	Superficial, narrowly ovoid or conoid, dark brown, 420–900 × 180–370 µm	Cylindrical, 300–600 × 4–5 µm with a 4 µm thick cap	Filiform, multi-septate, part-spores, 2–6 × 0.5–1 µm

Notes. ^1^Current study, ^2^Mongkolsamrit et al. (2018), ^3^Mains (1958).

## Supplementary Material

XML Treatment for Akanthomyces noctuidarum

XML Treatment for Akanthomyces pyralidarum

XML Treatment for Akanthomyces tortricidarum

## Supplementary materials

Supplementary material 1 MP tree Data type phylogenetic tree Explanation note Branches showing Maximum Parsimony bootstrap values. https://binary.pensoft.net/file/437786This dataset is made available under the Open Database License (http://opendatacommons.org/licenses/odbl/1.0/). The Open Database License (ODbL) is a license agreement intended to allow users to freely share, modify, and use this Dataset while maintaining this same freedom for others, provided that the original source and author(s) are credited.Arifah Nur Aini, Suchada Mongkolsamrit, Wijanarka Wijanarka, Donnaya Thanakitpipattana, J. Jennifer Luangsa-ard, Anto Budiharjo

Supplementary material 2 RAxML tree Data type phylogenetic tree Explanation note Branches showing Maximum Likelihood bootstrap values from RAxML. https://binary.pensoft.net/file/437787This dataset is made available under the Open Database License (http://opendatacommons.org/licenses/odbl/1.0/). The Open Database License (ODbL) is a license agreement intended to allow users to freely share, modify, and use this Dataset while maintaining this same freedom for others, provided that the original source and author(s) are credited.Arifah Nur Aini, Suchada Mongkolsamrit, Wijanarka Wijanarka, Donnaya Thanakitpipattana, J. Jennifer Luangsa-ard, Anto Budiharjo
