# Supplementary material for: Diversity of Akanthomyces on moths (Lepidoptera) in Thailand
Source: MycoKeys. 2020 Jul 30;71:1–22. doi: 10.3897/mycokeys.71.55126 (PMC7410849; doi:10.3897/mycokeys.71.55126)
Supplement: Supplementary material 1 — MP tree [file mycokeys-71-001-s001.docx]

Gibellula ratticaudata ARSEF1915

Hevansia arachnophila NHJ10469

Hevansia novoguineensis NHJ11923

Hevansia novoguineensis NHJ13161

Cordyceps nelumboides BCC2093

Hevansia cinerea NHJ3510

Akanthomyces websteri BCC23860

Gibellula sp. NHJ5401

Gibellula pulchra NHJ10808

Akanthomyces thailandicus TBRC7245

Akanthomyces sulphureus TBRC7248

Akanthomyces sulphureus TBRC7249

Akanthomyces waltergamsii TBRC7250

Akanthomyces waltergamsii TBRC7251

Akanthomyces farinosa CBS541.81

Akanthomyces coccidioperitheciata NHJ6709

Akanthomyces kanyawimiae TBRC7243

Akanthomyces kanyawimiae TBRC7242

Akanthomyces araneogenum GZUIF DX1

Akanthomyces araneogenum GZUIF SN1

Akanthomyces araneogenum GZUIF DX2

MY00952 02

MY06508

MY04235 02

MY02754

MY02910

MY05692

MY02898

MY03770

MY05935

MY02772

MY09590

Akanthomyces aculeatus HUA186145

Akanthomyces aculeatus TS772

Akanthomyces tuberculatus HUA186131

Akanthomyces attenuatus CBS402.78

Akanthomyces muscarius CBS455 70C

Akanthomyces muscarius CBS470 73

Akanthomyces muscarius CBS455 70B

Akanthomyces lecanii CBS101247

Isaria farinosa OSC111005

Isaria farinosa OSC111006

Samsoniella aurantia TBRC7271

Samsoniella aurantia TBRC7272

Isaria sp. spat09.050

Isaria sp. spat09.051

Samsoniella inthanonensis TBRC7915

Samsoniella inthanonensis TBRC7916

Beauveria acridophila HUA179221

Beauveria acridophila MCA1181

Beauveria brongniartii BCC16585

Beauveria brongniartii ARSEF617

Beauveria bassiana ARSEF1564

Beauveria caledonica ARSEF2567

Beauveria blattidicola MCA1727

Beauveria blattidicola MCA1814

Beauveria pseudobassiana ARSEF3405

Beauveria malawiensis ARSEF7760

Cordyceps fumosorosea CBS375.70

Cordyceps fumosorosea CBS107.10

Cordyceps coleopterorum CBS110.73

Cordyceps lepidopterorum TBRC7263

Cordyceps lepidopterorum TBRC7264

Cordyceps bifusispora spat08.129

Cordyceps bifusispora spat08.133.1

Cordyceps blackwelliae TBRC7253

Cordyceps blackwelliae TBRC7254

Cordyceps blackwelliae TBRC7255

Cordyceps cf. ochraceostromata ARSEF5691

Cordyceps tenuipes TBRC7265

Cordyceps tenuipes TBRC7266

Cordyceps takaomontana BCC12688

Cordyceps farinosa CBS111113

Cordyceps cateniannulata TBRC7258

Cordyceps ninchukispora spat08.115

Cordyceps ninchukispora spat09.021

Verticillium sp. CBS101284

Cordyceps caloceroides MCA2249

Cordyceps caloceroides QCNE186715

Cordyceps rosea spat09.053

Cordyceps kyusyuensis EFCC5886

Cordyceps militaris OSC93623

Cordyceps javanica TBRC7259

Cordyceps javanica TBRC7260

Cordyceps amoenerosea CBS107.73

Cordyceps amoenerosea CBS729.73

Lecanicillium psalliotae CBS101270

Lecanicillium psalliotae CBS532.81

Ascopolyporus polychrous

Ascopolyporus villosus ARSEF6355

Blackwellomyces cardinalis OSC93609

Blackwellomyces cardinalis OSC93610

Lecanicillium antillanum CBS350.85

Egyodontium aranearum CBS309.85

Torrubiella wallacei CBS101237

Simplicillium lamellicola CBS116.25

Simplicillium obclavatum CBS311.74

Simplicillium lanosoniveum CBS704.86

Simplicillium lanosoniveum CBS101267

OUT Purpureocillium lilacinum CBS284.36

OUT Purpureocillium lilacinum CBS431.87

100

92

100

70

92

73

55

53

100

88

95

100

94

87

85

98

75

99

100

100

100

84

80

79

96

93

70

100

99

90

51

100

53

57

100

89

53

100

57

85

77

77

100

100

92

92

98

74

83

81

86

88

100

66

97

73

93

100

65

65

98

100

100

52

68

53

100

100

100

100

100

98

63

55

95

100

58

100

100

100

100

100

78

72

100

100

100

100

98

Bootstrap consensus tree
