# Supplementary figures and images for: Diversity of Akanthomyces on moths (Lepidoptera) in Thailand
Source: MycoKeys. 2020 Jul 30;71:1–22. doi: 10.3897/mycokeys.71.55126 (PMC7410849; doi:10.3897/mycokeys.71.55126)

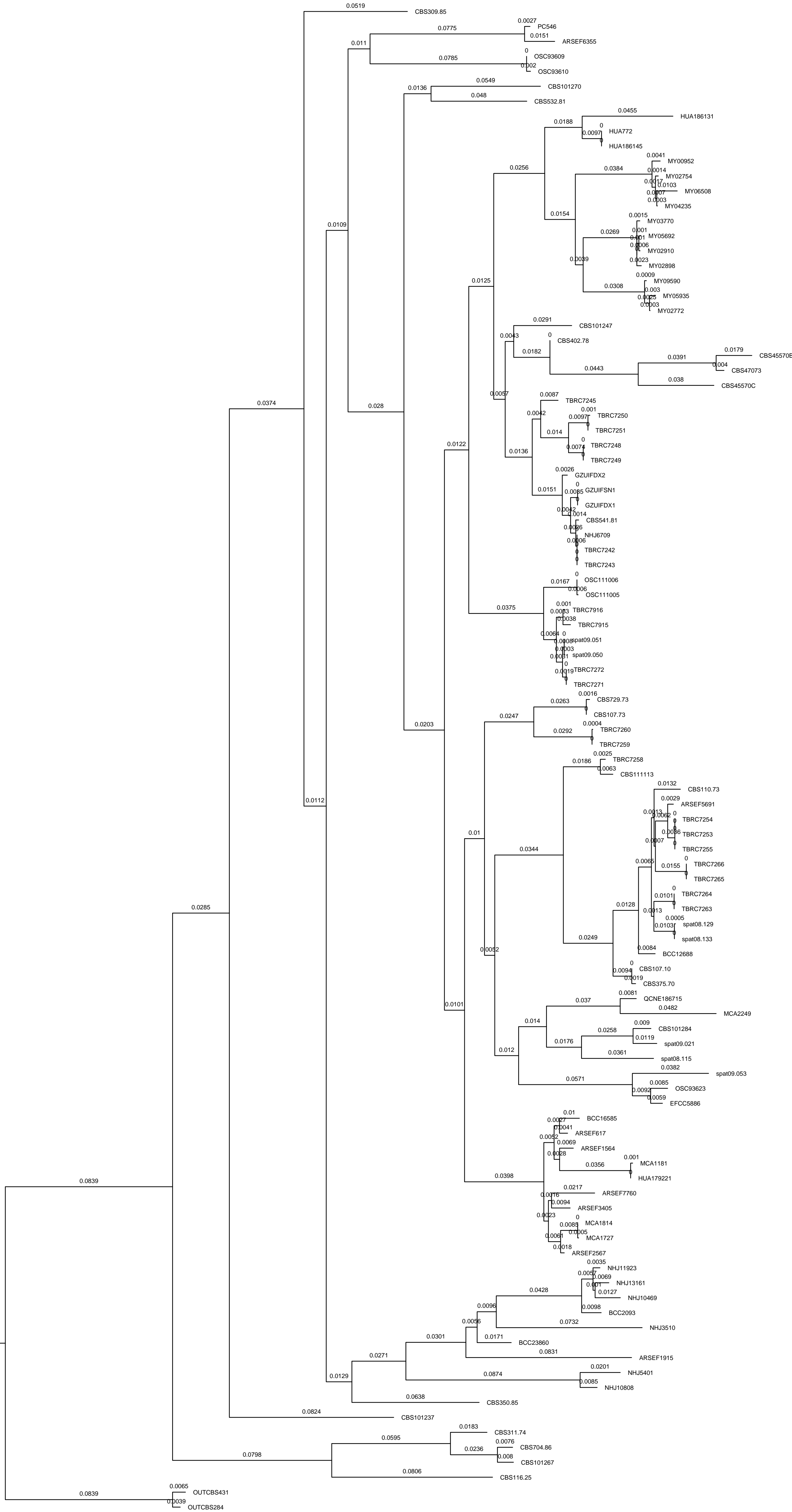

0.04

Supplement: Supplementary material 2 — RAxML tree [file mycokeys-71-001-s002.pdf]
